# Lymphocyte antigen 6 superfamily member D is a marker of urothelial and squamous differentiation: implications for risk stratification of bladder cancer

**DOI:** 10.1186/s40364-020-00232-1

**Published:** 2020-10-07

**Authors:** Nina Andersson, Johan Ohlsson, Sara Wahlin, Björn Nodin, Karolina Boman, Sebastian Lundgren, Karin Jirström

**Affiliations:** Division of Oncology and Therapeutic Pathology, Department of Clinical Sciences, Lund, Lund University, SE-221 85 Lund, Sweden

**Keywords:** Urothelial cancer, Urinary bladder cancer, Lymphocyte antigen 6 superfamily member D, Differentiation, Prognosis

## Abstract

**Background:**

Screening across a multitude of normal and malignant tissues revealed an enhanced expression of lymphocyte antigen 6 superfamily member D (LY6D) in squamous epithelium and urothelium, as well as in malignancies derived therefrom. The aim of this study was to further delineate the protein expression of LY6D in urothelial bladder cancer, with particular attention to its relationship with clinicopathological characteristics and patient outcome.

**Methods:**

Immunohistochemical expression of LY6D was assessed in tissue microarrays with urothelial bladder cancer tumours from three independent patient cohorts; one with transurethral resection of the bladder (TURB) specimens of mixed tumour stages from 110 consecutive cases, one with tumours of mixed stages from 260 incident cases in a population-based cohort, and one with paired TURB specimens, resected tumours and a subset of lymph node metastases from 145 patients with muscle-invasive bladder cancer (MIBC). Chi-square and non-parametric tests were applied to examine associations of LY6D expression with clinicopathological characteristics. Kaplan-Meier and Cox regression analyses were applied to examine 5-year overall survival (OS) and recurrence free survival (RFS) in relation to LY6D expression.

**Results:**

In the two cohorts with mixed stages, positive LY6D expression was denoted in 63 and 64% of the cases, respectively, and found to be significantly higher in low-grade and less invasive tumours. Negative LY6D expression was significantly associated with a reduced 5-year OS, although not independently of established prognostic factors. In the population-based cohort, LY6D expression was higher in tumours with squamous differentiation and lower in other variant histologies compared to pure urothelial tumours, and the association of LY6D expression with survival was somewhat enhanced after exclusion of the former. LY6D expression was generally lower in the MIBC cohort, and even more reduced in resected tumours compared to TURB specimens in patients who had not received neoadjuvant chemotherapy. There were no significant associations between LY6D expression and RFS, neither allover nor in relation to neoadjuvant chemotherapy.

**Conclusion:**

LY6D is a marker of urothelial and squamous differentiation that may add useful diagnostic and prognostic information to better guide the clinical management of bladder cancer, given that the presence of variant histology is taken into account.

## Background

Bladder cancer (BC) is the tenth most common malignancy in the world, with incidence and mortality rates in men being approximately four times those of women [1]. The most prevalent histological type of BC is urothelial carcinoma, and only a few percent comprise primary squamous cell carcinoma, adenocarcinoma or other rare tumour variants [2]. The majority of newly diagnosed BC, approximately 75%, are non-muscle invasive (NMIBC), with tumour growth restricted to the urothelium or underlying lamina propria (pTa, pT1 or Tis), and approximately 25% display upfront invasion of the detrusor muscle (stages T2–4), hence being classified as muscle-invasive BC (MIBC) [3].

NMIBC constitutes a heterogenous group of tumours with a high risk of recurrence and a considerable risk of becoming muscle-invasive [4]. Tumour stage and histological grade are important prognostic factors in NMIBC, although all hitherto proposed classification systems come with a certain degree of interobserver variability [5, 6], and there is an obvious need for complementary prognostic biomarkers for improved risk stratification of NMIBC. MIBC, in contrast, can be considered an upfront aggressive disease, with a considerable risk of developing distant relapse, often leading to death, within a few years after radical cystectomy. This risk can be reduced by the use of neoadjuvant chemotherapy (NAC), most likely due to its effect on micro-metastases being present already at diagnosis [7]. However, as the response to NAC varices [8], there is a need to identify predictive biomarkers that can improve the identification of patients with MIBC who will benefit from NAC.

Genes encoding proteins of the lymphocyte antigen 6 (Ly6) superfamily are located on human chromosome 8, longer arm, band 24 (8q24), alongside the proto-oncogene *c-Myc*. They are either transmembranous or secreted proteins widely spread across diverse cell types, and often upregulated in malignant compared to normal tissue (reviewed in [9]). Screening across a multitude of normal and malignant tissues in the Human Protein Atlas revealed a tissue enhanced expression of LY6D, in particular at the protein expression level, in the esophagus, skin, tongue, and urinary bladder, as well as in cancers derived from squamous epithelium or urothelium, such as head and neck, cervical or urothelial cancer [10]. These findings are in line with earlier studies [11], and LY6D has also been proposed to be a sensitive marker of urothelial carcinoma [12]. However, the potential clinical significance of LY6D expression in BC has, to the best of our knowledge, not yet been described.

The aim of this study was therefore to further delineate the immunohistochemical expression of LY6D in tumours from three independent cohorts of urothelial BC, two with mixed stages and one with surgically resected MIBC, the latter including both TURB specimens, resected primary tumours and paired lymph node metastases. Particular emphasis was given to the associations of LY6D expression with clinicopathological characteristics and patient outcome.

## Methods

### Study cohort I

This cohort, previously described in detail [13], constitutes a consecutive series of all patients (*n* = 110) with a first diagnosis of urothelial BC having undergone TURB at Skåne University Hospital, Malmö, between October 2002 and December 2003. Histopathological re-evaluation of the tumours was carried out by a board-certified pathologist (KJ), and the tumours were classified according to the WHO grading system of 2004. Information on vital status was obtained from the Swedish Cause of Death Registry up until December 31st 2010.

### Study cohort II

The second cohort encompasses all incident urothelial BC in the prospective, population-based Malmö Diet and Cancer study registered up until December 31st 2010, as described earlier [14, 15]. In brief, out of a total number of 355 cases, including non-bladder tumours, tumour tissue microarrays (TMA) were constructed from 272 cases, and the corresponding numbers for tumours located in the bladder were 335 and 264, respectively. All tumours were histopathologically re-evaluated and classified according to the WHO grading system of 2004 by a board-certified pathologist (KJ), whereby variant histology was also denoted. Information on vital status was obtained from the Swedish Cause of Death Registry up until December 31st 2012.

### Study cohort III

This cohort consists of a previously described consecutive series of all patients who underwent TURB and ensuing cystectomy for MIBC at Skåne University Hospital, Malmö, between January 1st 2011 and December 31st 2014 [16]. In brief, a total number of 145 cases were included, and both TURB and cystectomy specimens could be retrieved from 135 cases. All tumours were histopathologically re-evaluated by a board-certified pathologist (KJ), and were classified according to the WHO grading system of 2004. Clinical data were acquired from medical records, and follow-up lasted from the date of MIBC diagnosis until either death or August 31, 2018. Prior BCG-treatment had been given in 13(9.0%) cases, NAC in 65 (44.8%) cases, and adjuvant chemotherapy in 12 (8.3%) cases.

### Tissue microarray construction

Tissue microarrays (TMA) were constructed with duplicate 1 mm cores from TURB specimens in Cohort I, with duplicate 1 mm cores from TURB or cystectomy specimens in Cohort II, and with triplicate 1 mm cores of primary tumour samples from both TURB and cystectomy specimens, as well from a subset of lymph node metastases (*n* = 27) from the cystectomy specimens in Cohort III. A semi-automated arraying device (TMArrayer, Pathology Devices, Westminster, MD, USA) was used and all tumour specimens were, when possible, taken from different donor paraffin blocks.

### Immunohistochemical staining and evaluation

Four μm TMA-sections were automatically pre-treated with the PT-link system (Agilent Technologies, Santa Clara, CA, USA), including deparaffinization, rehydration and antigen retrieval for 20 min at 97 °C using the EnVision™ FLEX Target Retrieval Solution (3-in-1) pH 9 (Agilent technologies), in 65 °C preheat mode. The slides were then stained in an Autostainer Plus (Agilent Technologies) with a polyclonal antibody (HPA024755, Atlas Antibodies, Bromma, Sweden), dilution 1:200 and incubation time 30 min. The specificity of the antibody to LY6D has been demonstrated in several types of assays in the Human Protein Atlas. The estimated percentage of LY6D positive tumour cells was manually evaluated in each TMA core by three independent observers (NA, JO and KJ), and categorized as 0 (negative), 1 (≤50% positive cells) or 2 (> 50% LY6D positive cells). The staining intensity was not taken into account. Cases with lost cores or an insufficient amount of tumour cells in the TMA cores, as well as cystectomy specimens with no remaining tumour or carcinoma in situ (CIS) only, were excluded from the analyses. The mean score was denoted for multiple cores.

### Statistical analysis

For analysis of differences in the distribution of categories of LY6D expression according to clinicopathological parameters, Chi-squared test and non-parametric tests were used for categorical and continuous variables, respectively. Wilcoxon signed rank test was used to determine differences in LY6D expression between paired TURB specimens, cystectomy specimens and lymph nodes. Kaplan-Meier analysis and the log rank test were used to compare overall survival (OS) and recurrence free survival (RFS) in strata according to LY6D expression. Unadjusted and adjusted hazard ratios (HR) for 5-year OS and RFS in relation to negative (0) vs positive (1–2) LY6D expression were calculated using Cox regression proportional hazards modelling. The adjusted model only included variables that were significant in the unadjusted model. All tests were two sided and *p*-values < 0.05 were considered significant. All statistical analyses were performed using IBM SPSS Statistics version 25 (SPSS Inc., Chicago, IL, USA). The figures were constructed using SPSS or GraphPad Prism version 8 (GraphPad Software, LA Jolla, CA, USA).

## Results

### Distribution of LY6D expression

**Fig. 1:**
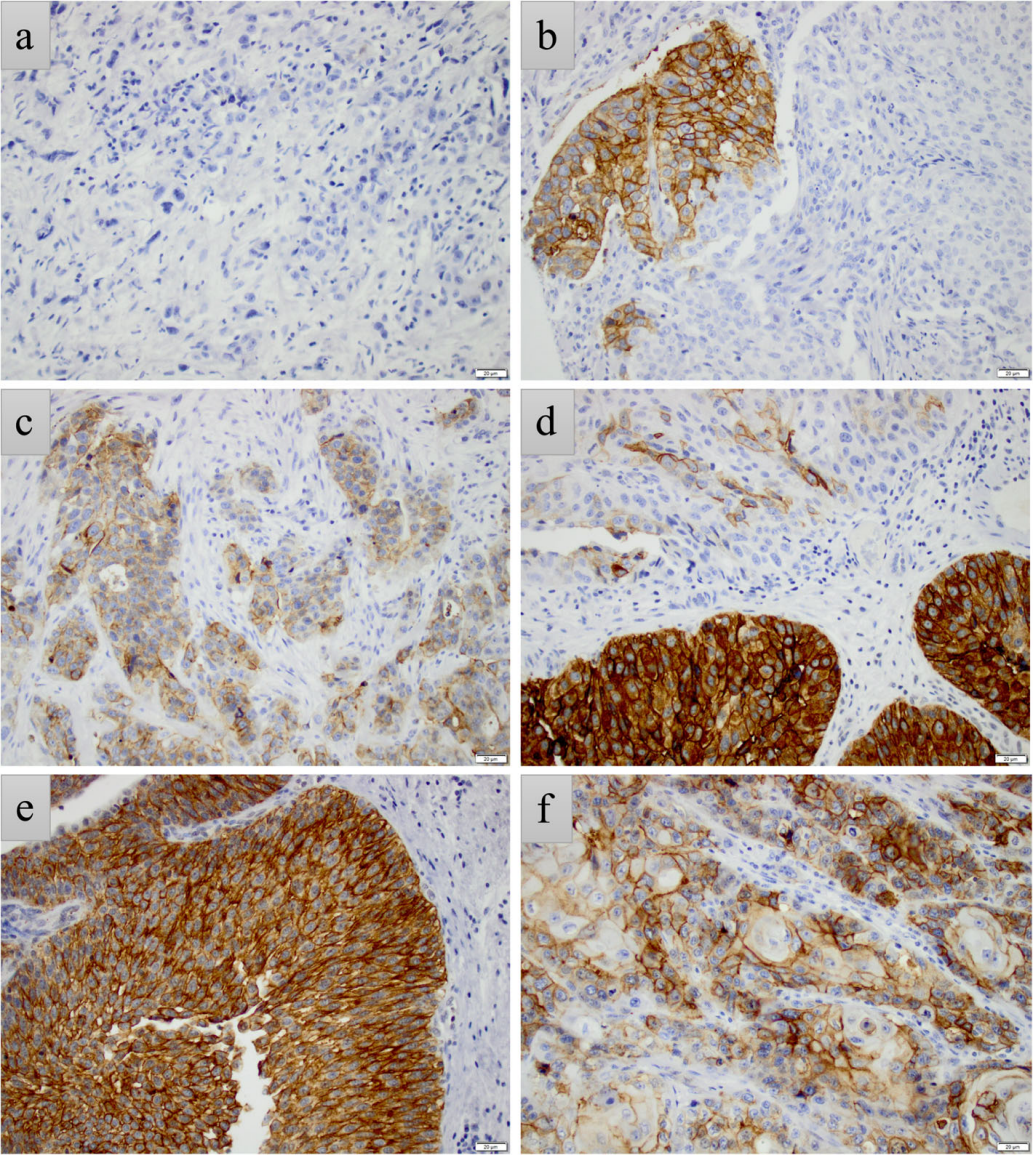
Patterns of LY6D expression. Sample immunohistochemical images of LY6D expression depicting (**a**) a negative pT2 high-grade tumour, (**b**) a pTa low-grade tumour with small positive foci in an otherwise negative tumour, (**c**) a pT2 high-grade tumour with positive LY6D expression in ≤50% of tumour cells, (**d**) a pT1 low-grade tumour with uniformly strong LY6D expression in the superficial part and weaker, more scattered expression in the invasive part (in total > 50% positivity), (**e**) a pTa low-grade tumour with uniformly strong expression, and (**e**) a pT4 high-grade tumour with squamous differentiation and positive expression in > 50% of tumour cells. All images 20x magnification, scale bar 20 μm

LY6D was expressed in the cytoplasm and cell membrane of tumour cells. No nuclear staining was observed. Sample immunohistochemical images are shown in Fig. 1. Areas with squamous differentiation were, as expected, LY6D positive. In some cases, LY6D expression was uniformly strong in the superficial part and more scattered in the invasive parts of the tumour. Of note, in line with previous observations, some tumours showed small foci of LY6D positive urothelial carcinoma cells within an otherwise completely negative setting [12]. No staining was denoted in stromal cells or immune cells.

**Fig. 2:**
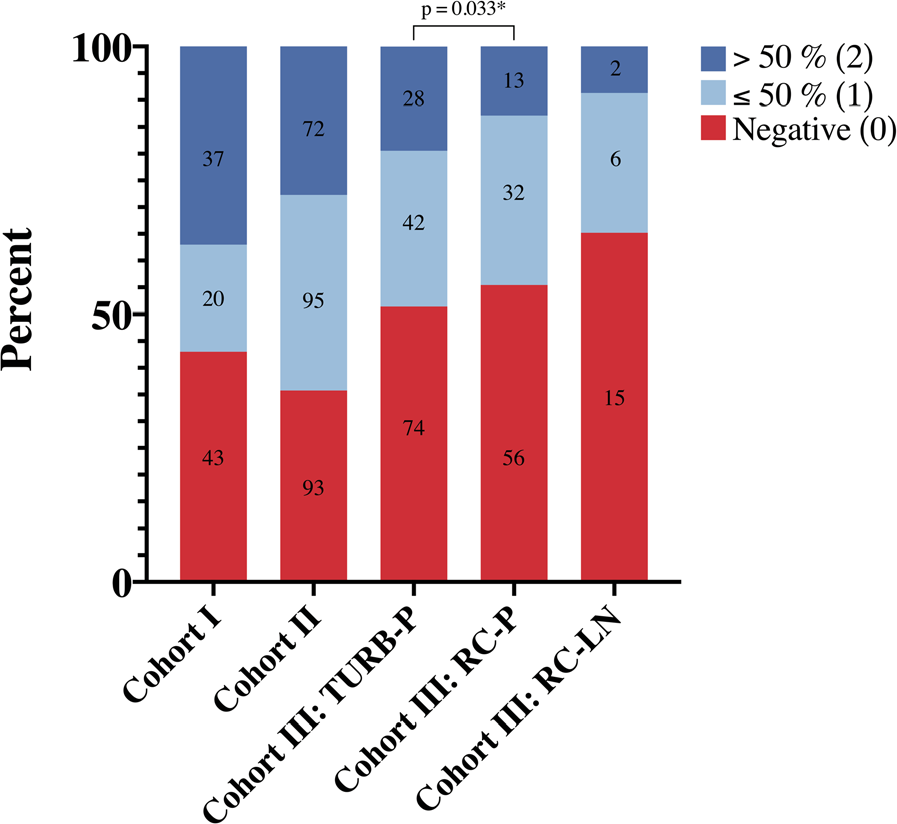
Distribution of LY6D expression. Bar charts visualizing the distribution of LY6D expression in the three cohorts, including paired primary tumours from TURB specimens and cystectomy specimens, respectively, and lymph node metastases in Cohort III. Colors represent percentages of each category of tumours with no LY6D expression, ≤50% positive cells, and > 50% positive cells, and the actual numbers within each category are also shown TURB-P: Primary tumour in TURB specimen, RC-P: Primary tumour in radical cystectomy specimen, RC-LN: Lymph node metastasis in radical cystectomy specimen. *P-value from Wilcoxon signed rank test for paired samples

The distribution of LY6D expression in the three study cohorts is shown in Fig. 2. In Cohort I, the expression of LY6D could be assessed in 100 (90.9%) cases, of which 37 (37%) were negative (0), 20 (20%) had ≤50% positive expression (1), and 43 (43%) had > 50% positive expression (2). In Cohort II, the expression of LY6D could be assessed in 260 (97.5%) cases, of which 93 (35.8%) were negative (0), 95 (36.5%) had ≤50% positive expression (1), and 72 (27.7%) had > 50% positive expression (2). In Cohort III, the expression of LY6D could be assessed in 144 (99.1%) TURB specimens, in 101 (74.8%) tumours from the cystectomy specimens, and in 23 (85.1%) lymph node metastases. Of the TURB specimens, 74 (51.4%) were negative (0), 42 (29.2%) had ≤50% positive expression (1), and 28 (19.4%) had > 50% positive expression (2). Corresponding numbers for tumours from the cystectomy specimens were 56 (55.4%) negative (0), 32 (31.7%) with ≤50% positive expression (1), and 13 (12.9%) cases with > 50% positive expression (2), and for lymph node metastases 15 (65.2%) negative (0), 6 (26.1%) with ≤50% positive expression (1), and 2 (8.7%) with > 50% positive expression (2). The expression of LY6D was significantly lower in cystectomy specimens compared to TURB specimens (*p* = 0.033), but did not differ significantly between lymph node metastases and primary tumours, neither in TURB nor in cystectomy specimens. When stratifying for NAC treatment, the difference in LY6D expression between TURB and RC specimens remained significant in cases who had not received NAC (*p* = 0.020), but not in cases who had received NAC (data not shown).

### Associations between LY6D expression and clinicopathological characteristics

The associations between LY6D expression and clinicopathological characteristics in Cohorts I and II are shown in Table 1. In both cohorts, LY6D expression was significantly higher in low-grade tumours, and in tumours with lower T-stages. In Cohort II, where variant histology had been denoted upon re-evaluation of the tumours, LY6D expression was found to be higher in tumours with squamous differentiation and lower in tumours with other variant histologies, e.g. nested, micropapillary, microcystic, glandular, sarcomatoid or giant cell, compared to classic urothelial tumours. Of note, in Cohort II, but not in Cohort I, LY6D expression was significantly higher in tumours of female patients than in male patients. There were no significant associations between LY6D expression and age at diagnosis in neither of the cohorts.

**Table 1:** Associations between LY6D expression and clinicopathological characteristics in Cohorts I and II

LY6D expression	Cohort I				Cohort II			
	0	1	2	*P-value*	0	1	2	*P-value*
n(%)	37 (37.0)	20 (20.0)	43 (43.0)		93 (35.8)	92 (36.5)	72 (27.7)	
**Age**								
Mean, median	73.3, 75,4	70.0, 67.0	68.0, 66.1	*0.174*	71.1, 71.2	71.2, 70.3	71.8, 72.7	*0.674*
range	40.1–88.5	46.0–84.3	39.3–89.9		52.0–85.0	52.9–86.6	51.2–86.8	
**Sex**								
Male	32 (86.5)	9 (45.0)	33 (76.7)	*0.382*	72 (77.6)	64 (67.4)	44 (61.1)	***0.023***
Female	5 (13.5)	11 (55.0)	10 (23.3)		21 (22.6)	31 (32.6)	28 (38.9)	
**pT-stage**								
pTa	11 (29.7)	9 (45.0)	12 (42.9)	***0.020***	26 (28.0)	49 (51.6)	39 (54.2)	***< 0.001***
pT1	10 (27.0)	4 (20.0)	10 (35.7)		32 (34.4)	29 (30.5)	20 (27.8)	
≥ pT2	16 (43.2)	7 (35.0)	6 (21.4)		35 (37.6)	17 (17.9)	13 (18.1)	
**Grade**								
Low	9 (24.3)	9 (45.0)	29 (67.4)	***< 0.001***	21 (22.6)	61 (64.2)	44 (61.1)	***< 0.001***
High	28 (75.7)	11 (55.0)	14 (32.6)		72 (77.4)	45 (35.8)	28 (38.9)	
**Histology**								
Classic	–	–	–		68 (73.1)	81 (85.3)	60 (83.3)	***0.016***
Squamous	–	–	–		4 (4.3)	3 (3.2)	7 (9.7)	
Other variant	–	–	–		21 (22.6)	11 (11.6)	5 (6.9)	
**Smoking history**								
Regularly	–	–	–		32 (34.4)	33 (34.7)	30 (41.7)	*0.184*
Occasionally	–	–	–		5 (5.4)	3 (3.2)	7 (9.7)	
Former	–	–	–		39 (41.9)	44 (46.3)	26 (36.1)	
Never	–	–	–		17 (18.3)	15 (15.8)	9 (12.5)	

*P*-value < 0.05 is considered significant

0 = Negative LY6D expression, 1 = ≤ 50% positive LY6D expression, 2 = > 50% LY6D expression

The associations between LY6D expression in primary tumours in TURB and cystectomy specimens, respectively, and clinicopathological characteristics in Cohort III are shown in Table 2. In this cohort consisting only of MIBC, and, hence, only high-grade tumours, LY6D expression in TURB specimens was significantly higher in T4 than in T2 tumours, but this association was not found in cystectomy specimens. In cystectomy specimens, high LY6D expression was significantly associated with higher N-stage and concomitant CIS. There were no significant associations between LY6D expression and any other clinicopathological characteristics. There were no significant associations between LY6D expression in TURB specimens and T-stage in cystectomy specimens, i.e. tumour regression, in strata according to NAC (data not shown).

**Table 2:** Associations between LY6D expression and clinicopathological characteristics in Cohort III

LY6D expression	TURB				Cystectomy			
	0	1	2	*P-value*	0	1	2	*P-value*
n(%)	74 (51.4)	42 (29.2)	28 (19.4)		56 (55.4)	32 (31.7)	13 (12.9)	
**Age**								
Mean, median	67.8, 69.7	69.6, 70.6	69.6, 72.5	*0.421*	69.7, 72.6	70.3, 70.6	74.0, 74.3	*0.288*
range	38.7–83.3	45.6–90.0	48.0–81.7		38.7–82.7	50.3–81.7	57.6–83.3	
**Sex**								
Male	57 (77)	37 (88.1)	20 (71.4)	*0.878*	44 (78.6)	26 (81.3)	9 (69.2)	*0.644*
Female	17 (23)	5 (11.9)	8 (28.6)		12 (21.4)	6 (18.8)	4 (30.8)	
**Preoperative T-stage**								
T2	44 (59.5)	20 (47.6)	12 (42.9)	***0.018***	26 (46.4)	16 (50.0)	7 (53.8)	0.677
T3	26 (35.1)	14 (33.3)	10 (35.7)		21 (37.5)	14 (43.8)	4 (30.8)	
T4	4 (5.4)	8 (19.0)	6 (21.4)		9 (16.1)	2 (6.3)	2 (15.4)	
**Postoperative T-stage**								
pT0/pTa/CIS only	26 (35.1)	14 (33.3)	4 (14.3)	*0.093*	7 (12.5)^a^	5 (15.6)	2 (15.4)	0*.349*
pT1	7 (9.5)	4 (9.5)	2 (7.1)		6 (10.7)	4 (12.5)	1 (7.7)	
pT2	14 (18.9)	8 (19.0)	7 (25.0)		11 (19.6)	6 (18.8)	3 (23.1)	
pT3	19 (25.7)	10 (23.8)	13 (46.4)		20 (35.7)	14 (43.8)	7 (53.8)	
pT4	8 (10.8)	6 (14.3)	2 (7.1)		12 (21.4)	3 (9.4)	0 (0.0)	
**N-stage**								
N0	51 (68.9)	31 (73.8)	20 (71.4)	*0.818*	31 (55.4)	23 (71.9)	11 (84.6)	***0.017***
N1	8 (10.8)	5 (11.9)	3 (10.7)		7 (12.5)	3 (9.4)	2 (15.4)	
N2	8 (10.8)	4 (9.5)	1 (3.6)		10 (17.9)	2 (6.3)	0 (0.0)	
N3	7 (9.5)	2 (4.8)	4 (14.3)		8 (14.3)	4 (12.5)	0 (0.0)	
**LVI in cystectomy**								
No	46 (93.9)	26 (89.7)	23 (100.0)	*0.448*	44 (89.8)	26 (92.9)	12 (100.0)	*0.253*
Yes	3 (6.1)	3 (10.3)	0 (0.0)		5 (10.2)	2 (7.1)	0 (0.0)	
*Missing*	*25*	*13*	*5*		*7*	*4*	*1*	
**CIS in TURB specimens**								
Not found	69 (93.2)	34 (81.0)	26 (92.9)	*0*.532	49 (87.5)	28 (87.5)	13 (100.0)	*0.299*
Found	5 (6.8)	8 (19.0)	2 (7.1)		7 (12.5)	4 (12.5)	0 (0.0)	
**CIS in cystectomy specimens**								
Not found	60 (81.1)	34 (81.0)	25 (89.3)	*0.396*	40 (71.4)	25 (78.1)	13 (100.0)	***0.039***
Found	14 (18.9)	8 (19.0)	3 (10.7)		16 (28.6)	7 (21.9)	0 (0.0)	
**NAC**								
No	39 (52.7)	21 (50.0)	19 (67.9)	*0.262*	40 (71.4)	22 (68.8)	10 (76.9)	*0.840*
Yes	35 (47.3)	21 (50.0)	9 (32.1)		16 (28.6)	10 (31.3)	3 (23.1)	

^a^Cystectomy specimens with tumour stage pT0 were excluded from the analysis

Correlations with grade were not analyzed since all 145 TURB tumours were high-grade and

only 3/104 assessable cystectomy tumours were low-grade

Correlations with M-stage were not analyzed since only 3 cases were M1

*CIS* Carcinoma in situ, *NAC* neoadjuvant chemotherapy

0 = Negative LY6D expression, 1 = ≤ 50% positive LY6D expression, 2 = > 50% LY6D expression

*P*-value < 0.05 is considered significant

### Associations between LY6D expression and clinical outcome

**Fig. 3:**
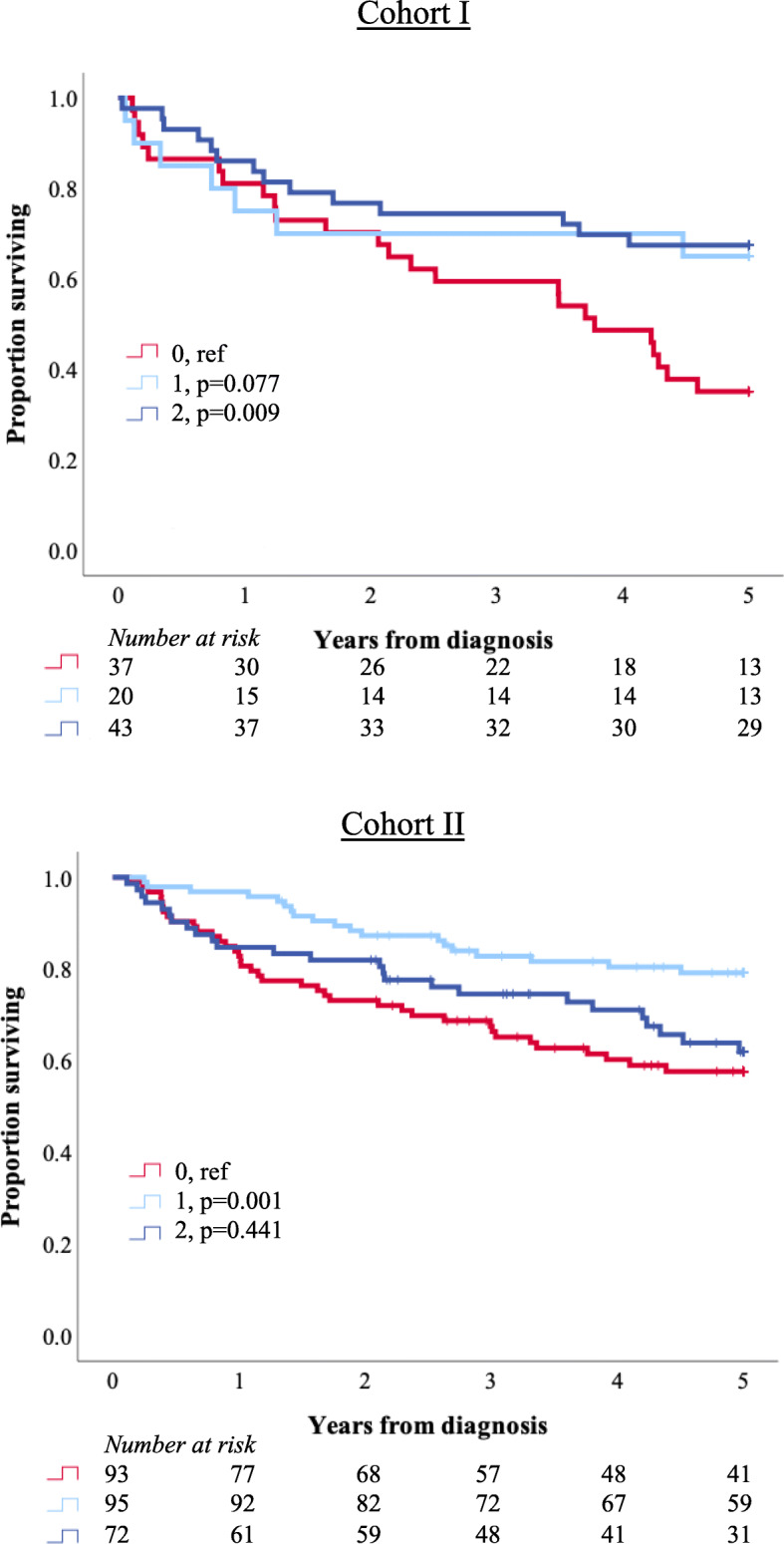
Survival according to LY6D expression in Cohort I and II. Kaplan-Meier analyses of 5-year overall survival in strata according to negative (0), ≤50% positive (1) and > 50% positive (2) LY6D expression primary tumours in Cohort I and Cohort II, respectively

**Fig. 4:**
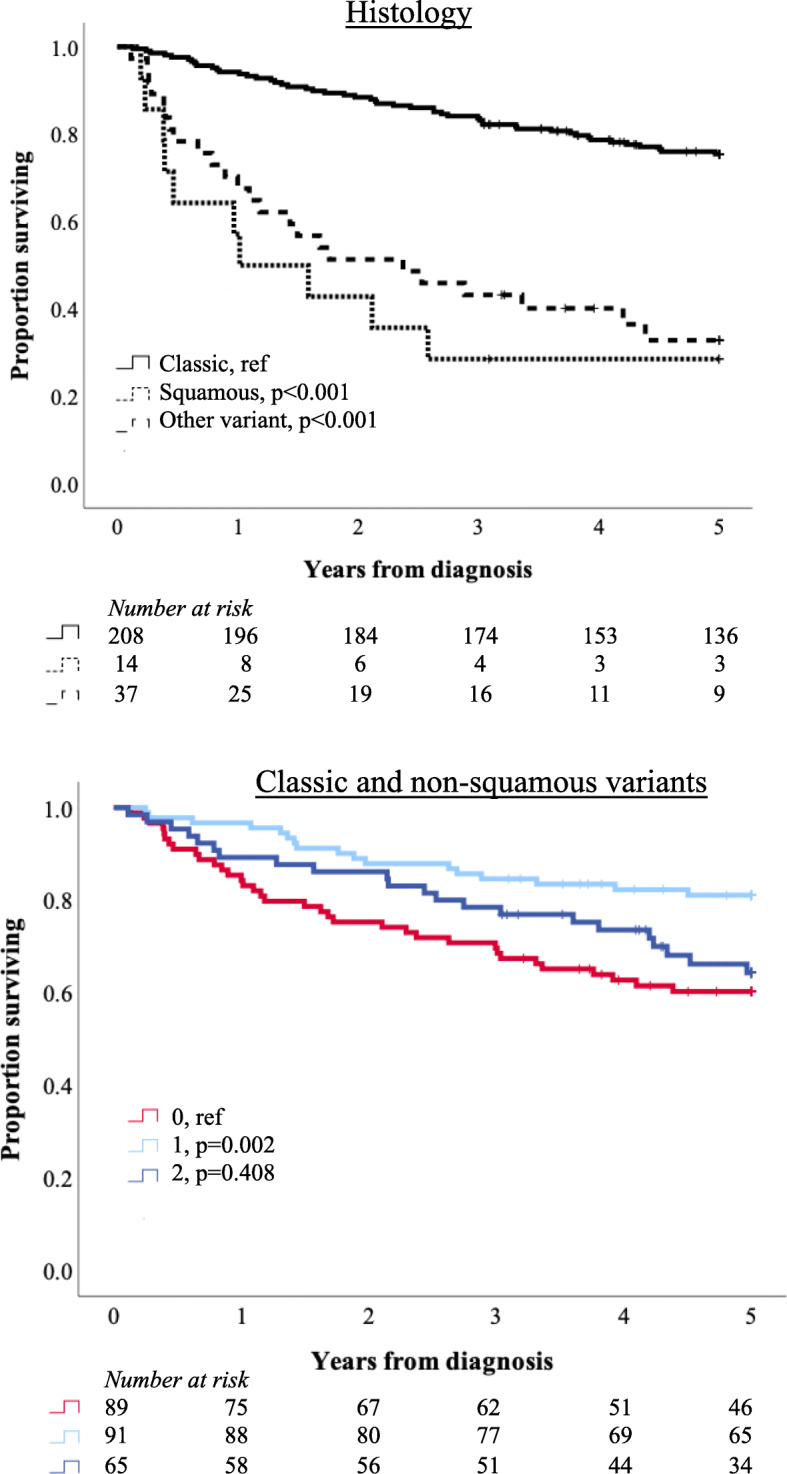
Survival according to histology and to LY6D expression in tumours without squamous differentiation. Kaplan-Meier analyses of 5-year overall survival according to histological type in Cohort III; classic urothelial tumours, tumours with squamous differentiation, and other variant histologies, as well as in strata according negative (0), ≤50% positive (1) and > 50% positive (2) LY6D expression after exclusion of tumours with squamous differentiation

Kaplan-Meier analyses of 5-year OS according to LY6D expression in Cohorts I and II are shown in Fig. 3. In both cohorts, the shortest survival was denoted in patients with LY6D negative tumours. Of note, in Cohort II, the best outcome was observed for patients with tumours displaying ≤50% LY6D positive expression, whereas there was no significant difference in 5-year OS between patients with negative tumours and patients with tumours displaying > 50% LY6D positive expression. As shown in Fig. 4, patients with variant histology tumours, in particular with squamous differentiation, had a significantly shorter survival than patients with urothelial tumours. The prognostic value of LY6D became more evident when tumours with squamous differentiation were excluded from the analysis, but did still not reach significance for the highest category (> 50%). LY6D expression was not prognostic in separate analyses of classic and any variant histologies, respectively (data not shown).

As shown in Table 3, any positive expression of LY6D was significantly associated with a prolonged 5-year OS in univariable but not in multivariable Cox regression analysis in both cohorts. Similarly, tumour grade was only prognostic in the univariable analysis in both cohorts, while age and T-stage remained independent prognostic factors in both cohorts. LY6D expression was not prognostic when grade was excluded from the model, and vice versa (data not shown). Variant histology, both squamous and other types, remained independent prognostic factors in Cohort II.

**Table 3:** Hazard ratios of death within five years according to LY6D expression in Cohorts I and II

	Cohort I			Cohort II		
		Univariable	Multivariable		Univariable	Multivariable
	n (events)	HR(95% CI)	HR(95% CI)	n (events)	HR(95% CI)	HR(95% CI)
**Age**						
Continuous	100 (45)	1.06 (1.03–1.09)	1.06 (1.02–1.09)	260 (82)	1.05 (1.02–1.08)	1.04 (1.01–1.08)
**Gender**						
Male	74 (33)	1.00	–	180 (59)	1.00	–
Female	26 (12)	1.15 (0.60–2.24)	–	80 (23)	0.88 (0.54–1.42)	–
**Stage**						
pTa	45 (7)	1.00	1.00	114 (16)	1.00	1.00
pT1	21 (14)	5.42 (2.18–13.47)	2.10 (0.69–6.37)	81 (24)	2.58 (1.37–4.87)	1.65 (0.80–3.57)
≥ pT2	34 (24)	7.98 (3.42–18.60)	3.39 (1.06–10.91)	65 (42)	8.14 (4.55–14.55)	3.47 (1.45–8.30)
**Grade**						
Low	47 (8)	1.00	1.00	126 (18)	1.00	1.00
High	53 (37)	6.35 (2.95–13.71)	2.26 (0.76–6.68)	134 (64)	4.47 (2.64–7.54)	1.44 (0.65–3.20)
**Histology**						
Classic				209 (48)	1.00	1.00
Squamous				14 (10)	6.12 (3.08–12.15)	2.39 (1.11–5.14)
Other variant				37 (24)	4.44 (2.71–7.27)	2.04 (1.16–3.59)
**LY6D expression**						
Negative (0)	37 (24)	1.00	1.00	93 (38)	1.00	1.00
Positive (1–2)	63 (21)	0.50 (0.25–0.81)	0.86 (0.47–1.60)	167 (44)	0.58 (0.37–0.89)	0.78 (0.48–1.25)

Only cases in which LY6D could be assessed were included in all analyses

Apart from LY6D expression, only factors with significant hazard ratios in the univariable analyses were

included in the multivariable analyses.0 = Negative LY6D expression, 1 = ≤ 50% positive LY6D expression, 2 = > 50% LY6D expression

**Fig. 5:**
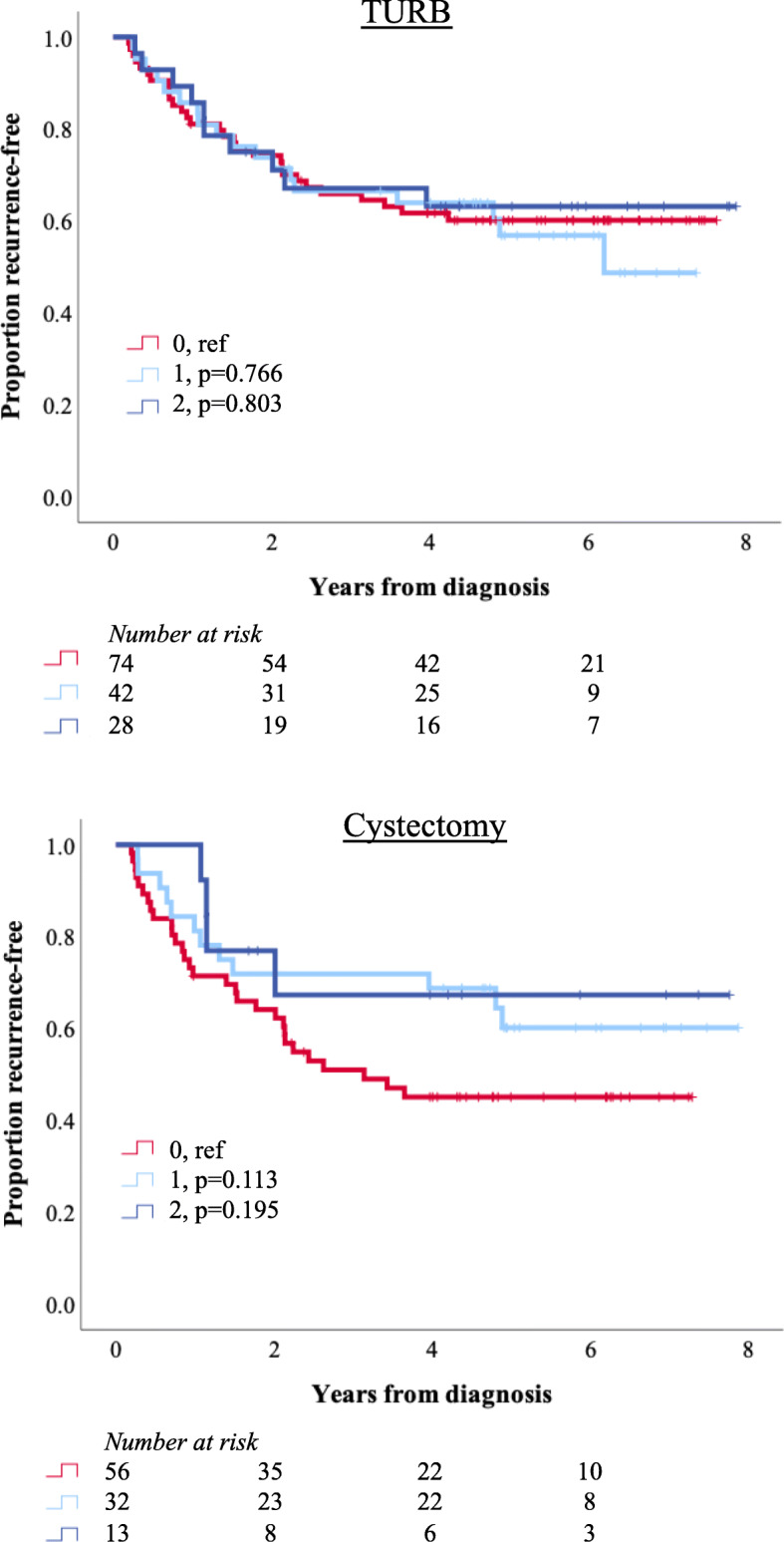
Survival according to LY6D expression in Cohort III. Kaplan-Meier analyses of 5-year overall survival in strata according to negative (0), ≤50% positive (1) and > 50% positive (2) LY6D expression in primary tumours in TURB specimens and cystectomy specimens in Cohort III

Kaplan-Meier analyses of RFS according to LY6D expression in TURB and resected primary tumours, respectively, in Cohort III showed that LY6D expression was not significantly associated with RFS, as illustrated in Fig. 5. Positive vs negative LY6D expression was not prognostic, neither in univariable nor multivariable Cox regression analysis (data not shown). LY6D expression was not prognostic in separate analyses in strata according to NAC (data not shown).

## Discussion

This study is, to our knowledge, the first to comprehensively describe the expression and clinicopathological correlates of LY6D in urothelial bladder cancer. The results, based on analyses of tumours from three independent patient cohorts, confirm a strong link between LY6D expression and differentiation grade. Moreover, LY6D expression was also found to be higher in tumours with squamous differentiation than in tumours with classic urothelial histology or in tumours with other variant histologies. The proportion of tumours being LY6D positive was quite similar in the two cohorts with both NMIBC and MIBC tumours, 63 and 64%, respectively, which is somewhat lower than in an earlier study on a smaller number of patients (*n* = 47) [12]. This difference may well be due to the fact that all analyses in the present study are based on tissue microarrays, which may render some false negative results given the inherent heterogenous staining pattern of LY6D denoted in some of the tumours. The larger proportion of tumours with ≤50% positive cells in Cohort II than in Cohort I may also be due to sampling bias.

In Cohort II, the presence of variant histology had been denoted upon histopathological re-evaluation, and in this cohort, LY6D expression was found to be higher in tumours with squamous differentiation and lower in tumours displaying other types of variant histology compared to classic urothelial tumours. Morover, variant histology in general, and squamous differentiation in particular, was found to be associated with a significantly shorter survival than urothelial histology. Consequently, when tumours with squamous differentiation, but not other variant histologies, were excluded from the survival analyses, the prognostic value of LY6D was found to be somewhat enhanced for the category with the highest expression, although still not reaching significance. LY6D expression was however not prognostic in separate analysis of tumours with classic urothelial histology only. Hence, the strong link between LY6D expression and squamous cell differentiation complicates the picture somewhat when it comes to the diagnostic and prognostic utility of LY6D expression in TURB specimens. LY6D has previously been suggested to be a suitable biomarker for differentiation between urothelial and prostate cancer, the latter being completely negative for LY6D expression [12]. Given the overall reduced expression of LY6D in MIBC, and in lymph node metastases, it is not likely to be a highly specific biomarker for identification of metastases from urothelial tumours, but might be helpful if the staining is positive and if squamous cell carcinoma can be excluded. It may also be a potentially useful prognostic biomarker, complementary to stage and grade, to more accurately identify NMIBC with a high risk of becoming muscle invasive, given that the presence of squamous differentiation is accounted for. A potential limitation to the survival analyses in the two cohorts with mixed tumour stages in the present study is that information on progression free or recurrence-free survival was not available. Overall survival after 5 years should however be considered an acceptable surrogate marker for disease progression, since all established prognostic factors were strongly associated with survival in both cohorts.

In some cases, there was a noticeable loss of LY6D expression in the more invasive parts of the tumour. These observations, together with the significant inverse associations of LY6D expression with T-stage in both mixed cohorts, are well in line with the lower proportion of 48% LY6D positive tumours denoted in the pure MIBC cohort. However, in contrast to the mixed cohorts, LY6D expression was found to be significantly higher in T4 compared to T2 tumours in the TURB specimens, but not in the cystectomy specimens. This observation suggests that LY6D might be more associated with cellular differentiation than with tumour invasiveness, although these features are interlinked. It should also be pointed out that MIBC is per definition a high-grade malignancy that differs from NMIBC in many aspects. Therefore, it is plausible that LY6D may exert other, possibly a preponderance of tumour promoting, biological effects in these tumours, similar to observations made in e.g. prostate cancer [17], or estrogen-receptor positive breast cancer [18]. On the other hand, in primary tumours from the cystectomy specimens, LY6D expression was inversely associated with N-stage and concomitant CIS, both being adverse prognostic factors.

While the proportion of LY6D negative cases was larger in lymph node metastases in the MIBC cohort, the expression did not differ significantly between paired samples of lymph node metastases and primary tumours, neither in TURB nor in cystectomy specimens. However, the finding of a significantly lower expression of LY6D in primary tumours from cystectomy specimens than from paired TURB specimens, in particular in patients who had not received NAC, merits some attention. Firstly, it must be pointed out that there were no indications of LY6D expression being prognostic in MIBC, neither its expression in TURB nor in cystectomy specimens, and the lacking association with prognosis was observed regardless of NAC. Moreover, there were no significant associations between LY6D expression and tumour regression, i.e. postoperative T-stage, in patients receiving NAC. Therefore, rather than assuming that patients not treated with NAC prior to cystectomy may be at higher risk of developing a more aggressive disease, reflected in a larger proportion of LY6D negative cells in the cystectomy specimens, it can be speculated that LY6D positive tumours may be more resistant to chemotherapy and therefore to a lesser extent eradicated after NAC. In support of this hypothesis, LY6D has indeed been shown to confer chemoresistance in head and neck squamous cell carcinoma [19, 20], and Rubinstein et al. demonstrated that LY6D was consistently up-regulated in colorectal cancer xenografts grown in mice upon treatment with irinotecan, and that treatment with a combination of LY6D-targeting antibody and irinotecan led to complete tumour regression [21]. Hence, the potential utility of LY6D as a target for neoadjuvant treatment of MIBC merits further study. It would also be of interest to examine whether LY6D is a predictive marker of chemoresistance in tumours from a randomized trial, as the real-world setting of the herein studied MIBC cohort does not allow for more than cautious speculations in this regard.

## Conclusions

LY6D is a marker of urothelial and squamous differentiation and the extended validation in the present study supports its potential clinical utility as a complementary diagnostic and prognostic biomarker for improved clinical management of patients with bladder cancer, given that the presence of variant histology is taken into account. It would also be of interest to address the role of LY6D in the context of chemotherapy response in future studies, or even whether inhibition of LY6D may provide therapeutic benefits for patients with MIBC.

## Data Availability

Part of the data generated in this study is included in the article. The raw data of LY6D receptor expression can be made available upon request. Patient and clinicopathological data in the different study cohorts cannot be made publicly available due to their content of identifiable human data. Requests to access the datasets should be directed to Karin Jirström.

## Abbreviations

BC: Bladder cancer
CIS: Carcinoma in situ
Ly6: Lymphocyte antigen 6
LY6D: Lymphocyte antigen 6 superfamily member D
MIBC: Muscle-invasive bladder cancer
NAC: Neoadjuvant chemotherapy
NMIBC: Non-muscle invasive bladder cancer
OS: Overall survival
RFS: Recurrence free survival
TMA: Tissue microarray
TURB: Transurethral resection of the bladder
